# Intravenous administration of adenosine triphosphate and phosphocreatine combined with fluoxetine in major depressive disorder: protocol for a randomized, double-blind, placebo-controlled pilot study

**DOI:** 10.1186/s13063-018-3115-4

**Published:** 2019-01-09

**Authors:** Yiyi Chen, Xiaomin Cao, Wensi Zang, Shanyong Tan, Chun-quan Ou, Xiaoyan Shen, Tianming Gao, Lianxu Zhao

**Affiliations:** 1 Department of Neurology, Zhujiang Hospital, Southern Medical University, Guangzhou, China; 2 Department of Neurobiology, School of Basic Medical Sciences, Southern Medical University, Guangzhou, China; 3 State Key Laboratory of Organ Failure Research, Department of Biostatistics, Guangdong Provincial Key Laboratory of Tropical Disease Research, School of Public Health, Southern Medical University, Guangzhou, China; 4 Department of Neurology, Shenzhen Hospital, Southern Medical University, Shenzhen, China

**Keywords:** Major depressive disorder, Adenosine triphosphate, Phosphocreatine, Fluoxetine, Fast-acting antidepressant, Randomized controlled trial

## Abstract

**Background:**

Major depressive disorder (MDD) is a common psychiatric disorder. With systematic antidepressant treatment, 50–75% of patients have a treatment response but require 4–6 weeks to have their symptoms alleviated. Therefore, researchers anticipate the development of novel fast-acting antidepressants. Previous studies have revealed that the decrease of bio-energetic metabolism may contribute to the occurrence of depression, while our team has found adenosine triphosphate (ATP) and phosphocreatine (PCr) to be fast-acting antidepressants in the depressed-animal model. ATP and PCr have already been widely prescribed clinically as energy supplements for cells. This will be the first clinical attempt of the intravenous administration of ATP and PCr combined with orally administered fluoxetine in MDD.

**Methods:**

This is a single-center, randomized, double-blind, placebo-controlled pilot study. A total of 42 patients will be divided randomly into three groups. Patients will receive an intravenous administration of ATP or PCr or saline twice daily combined with orally administered fluoxetine (20 mg/day) for the first 2 weeks and fluoxetine monotherapy for the following 4 weeks. Follow-up assessment will be completed at week 10. Feasibility outcomes will include percentages of patient eligibility, intention to use medication, willingness to participate, drug adherence, completion of the scheduled assessment, retention, drop-out, etc. Physical examination results, Side Effect Rating Scale, adverse events, results from blood tests, electroencephalogram, and electrocardiograph will be recorded for safety evaluation of the augmentation therapy. The trends of efficacy will be evaluated by the reduction rate of the Hamilton Depression Rating Scale, the mean change of the Clinical Global Impression Scale, and the Patients Health Questionaire-9 items.

**Discussion:**

In our study, ATP and PCr will be given by intravenous infusion. Thus patients will be hospitalized for the initial 2 weeks for safety concern. Hospitalization will be an impact factor for the recruitment, participation, drop-out, efficacy, results, etc. The evaluation of our feasibility outcomes, study setting, safety of augmentation therapy and possible efficacy trends among groups, will facilitate a full-scale trial design and sample size calculation.

**Trial registration:**

NCT03138681. Registered on 3 May 2017. First patient: 4 May 2017.

**Electronic supplementary material:**

The online version of this article (10.1186/s13063-018-3115-4) contains supplementary material, which is available to authorized users.

## Background

Major depressive disorder (MDD) is a long-lasting mental disturbance [1]. In China, the lifetime prevalence of MDD is approximately 3.3% [2]. It was accidentally observed that patients treated with reserpine, an antihypertension drug that depletes catecholamines, experienced depression, indicating a possible relationship between depletion of norepinephrine or 5-hydroxytryptamine in the brain and affective disorders [3]. Since then, the pharmacology of antidepressants (ADs) has generally been based on the classical monoaminergic hypothesis. To date, the selective serotonin reuptake inhibitors (SSRIs) have generally been selected when treating moderate to severe MDD.

Overall, with adequate first-line pharmacological treatment, 50–75% of patients experience symptom alleviation while the remaining exhibit no response to treatment. Generally, symptom alleviation requires 4–6 weeks after starting medication [4, 5]. Rapid response to treatment could improve patients’ adherence to regular drug intake and strengthen their confidence in overcoming depression, thus largely minimizing the risk of suicide. It may be true that the more progress made in the early treatment phase (first 2 weeks), the more favorable the future outcome that could be achieved [6]. Therefore, researchers anticipate the development of novel fast-acting ADs with minimal side effects.

One major strategy revolves around the modulation of the glutamatergic system. Ketamine, a non-competitive *N*-methyl-D-aspartate (NMDA) receptor antagonist, has attracted considerable attention. However, concern regarding potential drug abuse and psychotomimetic manifestations related to NMDA receptor blockage have prevented its broad clinical administration, particularly in individuals with a history of psychosis [7, 8].

Depression has been considered a syndrome rather than a single disease because even though patients exhibit the same clinical manifestations, the underlying etiology is highly heterogeneous [9]. We intend to identify an AD predominantly based on “the dysfunctional energy homeostasis hypothesis.”.

In multiple studies conducted on animal models of depression, several proteins involved in glycolysis and the tricarboxylic acid cycle, such as mannose-6-phosphate isomerase, glyceraldehyde-3-phosphate dehydrogenase, pyruvate kinase, and lactate dehydrogenase, as well as fumarate hydratase, aconitase, and the malic enzymes ME1 and ME2, have shown increased expression in cerebellar samples. Nevertheless, several metabolites related to energy metabolism (e.g., creatine, succinic acid, and glucose-1-phosphate) have been shown to be decreased both in the cerebellum and the prefrontal cortex (PFC), suggesting that energy deficiency with depression leads to compensatory upregulation of the enzymes associated with energy metabolism [10–12]. Positron emission tomography of patients with MDD, reflecting the blood flow and glucose metabolic rate in the brain, suggests reduced blood flow or glucose metabolism in the PFC, anterior cingulate cortex, and caudate nucleus. Conversely, in patients with bipolar disease, the reduced metabolic rate increases when transitioning from depression to the manic state [13]. The studies have demonstrated that altered metabolism in the PFC-limbic-striatal region could be normalized after alleviating clinical symptoms by both ADs treatment and non-pharmaceutical therapy [14, 15]. Bremner et al. found that when patients with MDD were in a remission state after SSRI treatment and underwent tryptophan-depletion procedures, 7 of 21 patients experienced a depressive relapse, accompanied by decreased or unchanged cerebral metabolism in the thalamus and middle frontal gyrus, whereas those without relapse displayed unaltered cerebral metabolism. Moreover, there is a significant inverse correlation between the metabolic state and the Hamilton Depression Rating Scale (HAMD) score [16].

Mitochondria are the powerhouses generating adenosine triphosphate (ATP). The tremendous energy demand of neuronal activities is provided by oxidative phosphorylation (OXPHOS) inside the mitochondria. The mitochondrial respiratory chain polypeptides are comprised of five respiratory chain enzyme complexes and two electron carriers, namely, coenzyme Q10 (CoQ10) and cytochrome.

Studies around MDD and mitochondrial activity or ATP synthesis have been conducted and proved to be relevant [17–22]. Approximately 54% of subjects diagnosed with a mitochondrial disease have comorbid MDD. “Mitochondrial cocktails” for the treatment of mitochondrial diseases, CoQ10/creatine in particular, also exert AD-like effects [23]. When treated with CoQ10 for 21 days, rats exposed to chronic restraint stress showed improvement both in the forced swimming test and the open-field test [24]. Clinically, a 4-week treatment of CoQ10 in geriatric bipolar depression resulted in a significant reduction in Montgomery Asberg Depression Rating Scale scores [25]. In an 8-week study of oral treatment with creatine, female adolescents with SSRI-resistant MDD showed a steady decrease in Children’s Depression Rating Scale-Revised (CDRS-R) scores and a significant increase in PCr in the brain on ^31^P magnetic resonance spectroscopy [26].

The limited reservoir of ATP in the brain demands a high re-synthesis rate to fuel vast energy requirements, accounting for approximately 20% of all energy production. In contrast, PCr, stored abundantly in the brain, transiently transfers high-energy phosphoryl bonds to form ATP through the activity of creatine kinase (CK). Thus, the continuous supply of the high-energy phosphoryl bonds by PCr appears to contribute to the dynamic equilibrium of ATP consumption and ATP synthesis [27].

In our preclinical studies, the ATP levels were significantly decreased in artificial cerebral spinal fluid, as well as in the interstitial fluid in the hippocampus and PFC, collected in susceptible mice but not in unsusceptible mice after chronic social-defeat stress. Lateral intracerebroventricular injection of ATP reduced total immobility time in the forced swimming test. Furthermore, 7-day intraperitoneal injections of ATP yielded AD-like effects to an extent similar to that of 28-day treatment with imipramine [28]. Additionally, intraperitoneal injection of PCr in the depressed rats at multiple dosages (31.25, 62.5, 125, 250 mg/kg) achieved remarkable AD effects, with 125 mg/kg PCr producing the best efficacy.

Together, the researches highlight a pivotal connection between reduced levels of ATP/PCr and depression. For this reason, we hypothesized that ATP and PCr may be potential fast-acting ADs in the treatment of MDD.

### Research objectives

Our pilot study aims to evaluate the feasibility and safety to conduct a full-scale trial of intravenous (IV) administration of ATP and PCr combined with orally administered fluoxetine to patients with moderate to severe MDD. The preliminary exploration of the AD effect of augmentation therapy over fluoxetine alone might allow us to obtain clinical insights beyond the results from preclinical animal studies.

### Trial design

**Fig. 1:**
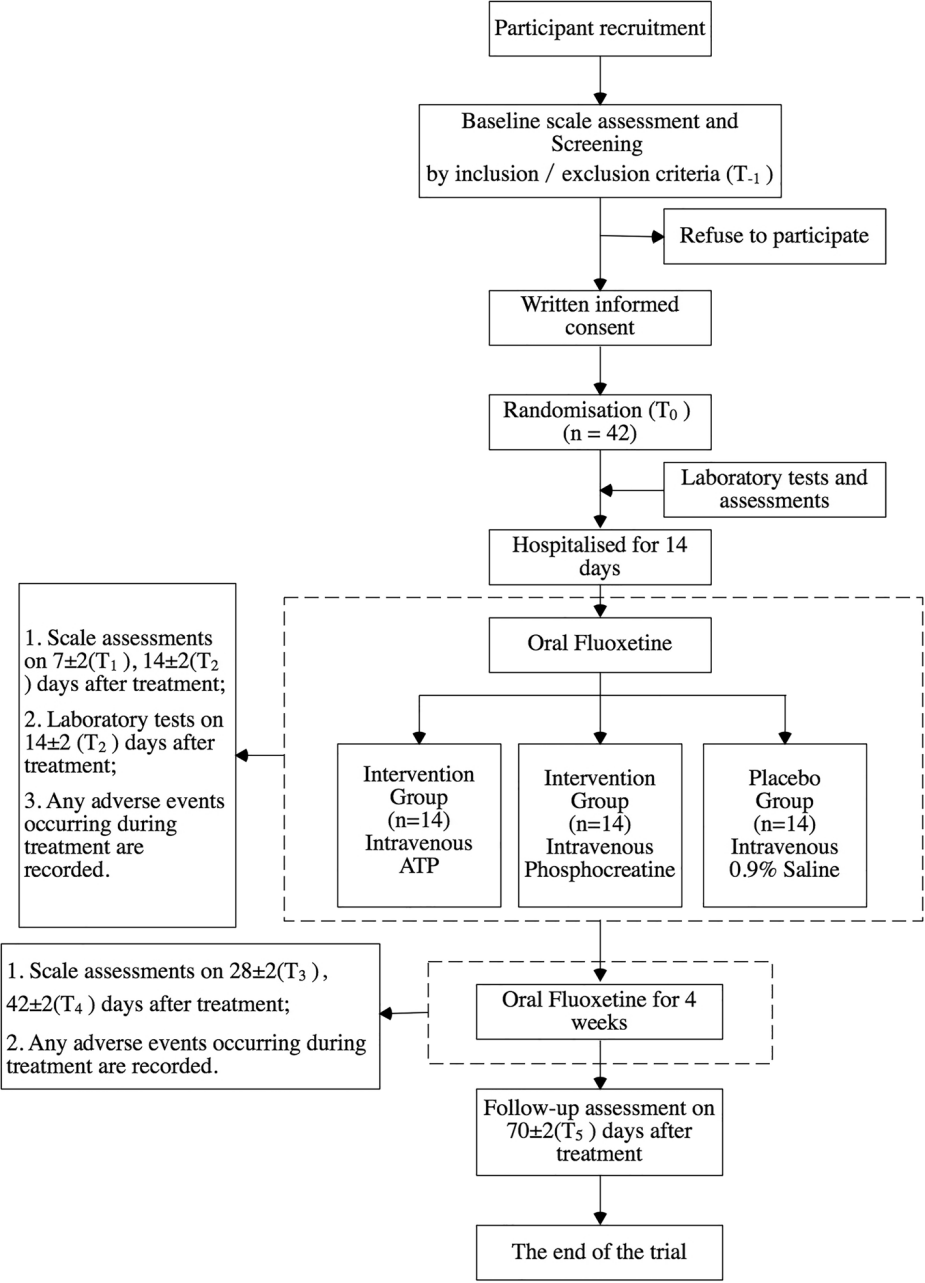
Study process

This trial is designed as a single-center, randomized, double-blind, placebo-controlled clinical pilot study. An overall summary of our study process is shown in Fig. 1.

## Methods: Participants, interventions, and outcomes

### Study setting

The study will be conducted at Zhujiang Hospital, Southern Medical University, Guangzhou, China. Patients will be assessed and hospitalized in the Neurology Department of the hospital.

### Eligibility criteria

#### Inclusion criteria

Patients eligible for the trial must meet all the following criteria:

- Firstly diagnosed with moderate to severe MDD, according to the *Diagnostic and Statistical Manual of Mental Disorders, Fifth Edition* (DSM-V)
- HAMD-24 total score ≥ 20
- Men and women aged between 18 and 65 years old; and
- Voluntary and written informed consent

#### Exclusion criteria

Patients meeting any of the following criteria will be excluded:

- Enrolled in any clinical trials in the previous 4 weeks
- Previously diagnosed with MDD or a lifetime history of schizophrenia
- Experienced traumatic events in the previous 6 months and diagnosed with posttraumatic stress disorder
- Pregnant, breastfeeding or planning to do so
- Severe physical diseases, such as severe dementia, aphasia, consciousness disturbance, and deafness, which could interfere with the judgment of depression
- Other psychiatric disorders such as schizophreniform disorder, schizotypal (personality) disorder, and bipolar disorder
- Severe medical complications, such as hepatic, cardiac, pulmonary or renal dysfunction
- Neurological disorders, e.g., epilepsy, acute stroke, multiple sclerosis, and malignant cerebral diseases
- Inflammatory diseases, including autoimmune disorders
- Current immunosuppression treatment or intake of antiarrhythmic, antidiabetic drugs or tryptophan agents
- A history of substance-related or addictive disorders in the previous 6 months;
- Suicide attempts; and
- Unwillingness to participate in, or having difficulties, completing our study

### Interventions

Low-dose IV infusion of ATP (20–40 mcg/kg/min) has been shown to be safe in multiple clinical trials [29, 30]. Therefore, IV administration of 100 mg ATP (40 mcg/kg/min) twice daily was chosen in this study. PCr has been used in muscle hypotonotrophy, acute and chronic ischemic heart disease, heart failure conditions, and ischemic stroke. Clinically, no serious side effects have been reported for the IV administration of 1–2 g of PCr daily for the treatment of the above-mentioned diseases [31]. Besides, clinical studies have reported the safety of 3–5 g/day of orally administered creatine for MDD [32–34]. Our animal studies found that intraperitoneal injection of 125 mg/kg PCr in depressed rats producing the best efficacy; thus, IV administration of PCr at 1 g (125 mg/kg × 0.08 × 60 kg) twice daily was chosen in this study [35].

Enrolled patients will be hospitalized for the initial 14 days. All patients will receive one fluoxetine tablet (Eli lilly and company, State Food and Drug Administration (SFDA) approval No. J20160029, 20 mg per day, at 08:00). For the ATP and PCr groups, adenosine disodium triphosphate (Guangzhou Baiyunshan Tianxin pharmaceutical corporation, SFDA approval No. H44023293, 100 mg, 10 ml) or sodium phosphocreatine (Harbin Laiboten pharmaceutical corporation, SFDA approval No. H20054352, 1 g in 10 ml of 0.9% saline) diluted in 100 ml of 0.9% saline will be administered intravenously twice daily (at 08:00 and 16:00). IV administration should be completed within 40 min (40 drops per min) of time of dilution. In the control group, patients will receive 110 ml of 0.9% saline in equivalent procedures. In total, we will provide continuous orally administered fluoxetine therapy for 6 weeks, and intravenously administered medical treatment will proceed during hospitalization.

### Discontinuation criteria

If participants meet one of the following criteria, the study drugs will be withdrawn:

- ■ The drug-related utilization ratio (number of actual tablets taken/number of planned tablets × 100%) is less than 80% or greater than 120%
- ■ The risks in continuing to participate outweigh the benefits of our study due to the appearance or exacerbation of other diseases
- ■ The aggravation of patients’ mental conditions, such as evolution into severe depression or manic episodes, a high risk of suicide attempts or suicidal acts, which would require altering medical treatment during the study period
- ■ Severe side effects, severe adverse events; and
- ■ Anticipated or accidental pregnancy
- ■ Patients can leave our trial at any time for any reason
- ■ The reason for withdrawing should be recorded on the Case Report Form (CRF)

### Combination therapies

- ■ We prefer that enrolled subjects with mild sleep difficulties not receive any sleeping pills. Zolpidem, zopiclone or estazolam will be prescribed for patients with severe insomnia disorders
- ■ Enrolled subjects with other physical illnesses will continue with their original treatment plan
- ■ Considering the precision of our collected clinical outcome data, antipsychotics, ADs or mood stabilizers (e.g., sodium valproate, lithium carbonate) will not be allowed to be prescribed during the study period. Similarly, any drugs that could have interactions with our study drugs, such as antiarrhythmic or antidiabetic drugs or tryptophan, will not be administered either
- ■ The participants’ treatment history and concomitant treatments should be recorded on the CRF

### Adherence

Before obtaining written informed consent, the patients will be comprehensively briefed on both the importance of conducting this clinical study and the value of their participation. Specifically, detailed information on our study procedure, its potential risks and its effectiveness will be conveyed to the subjects. Moreover, the economic burden of taking ADs can be partially alleviated, and subsidies, supported through funding, will be paid after they accomplish their follow-up assessments. During hospitalization, study drugs and doses will be monitored by the research nurse. Thus, medication compliance will be guaranteed. After discharge, the drug regimen and follow-up arrangement will be explained to the patients. The physician-patient relationship will be built on mutual respect and trust.

### Outcomes

#### Study feasibility outcomes

Feasibility evaluation includes

**Table:** 

Outcomes	
Recruitment period	
Feasibility outcomes	Our pilot study will be conducted in a general hospital. The difficulties while recruiting will be recorded and discussed in the final report. Regarding our study inclusion criteria, moderate to severe MDD patients with HAMD-24 ≥ 20 can be enrolled. Some of our eligible patients might be inclined to choose non-pharmacological treatment. The percentage of eligible patients refusing to participate in our study. Their reason will be recorded on the recruitment document and discussed in the final report. The percentage of eligible patients willing to participate in our study. Their reason will be recorded on the recruitment document and discussed in the final report
recruitment difficulties	
eligibility rate	
intention to use medication	
refusal rate	
participation rate	
Study period	
Feasibility outcomes	While hospitalized, the drug compliance will be guaranteed by bedside nurses. The assessment of adherence rate will follow schedule and their reason for not taking drugs will be recorded. (see Fig. 2). The percentage of enrolled patients withdrawn before completion of our study. Their reason will be recorded on the Case Report Form (CRF). If “yes,” the researcher should list the name/dosage/duration, and explain its reason for use on the CRF. After study period, the percentage of enrolled patients willing to receive medical advice from our clinical researcher. The occurrence rate of side effects or the adverse events. Test the conduction of our blinding method
adherence	
withdrawn	
concomitant therapy	
retention	
side effects/adverse events	
blinding	
Overall	
Feasibility outcomes	At any time of their participation, enrolled patients will be encouraged to report their suggestion or feeling to researcher. Also, they will receive a questionnaire questioning their perceptions toward the medical environment, treatment response, sense of security and suggestion while hospitalized. It will be recorded and discussed in final report. The appropriateness of the implementation of our study scheme and it will be discussed in final report
participation/hospitalization feedback	
study scheme	

### Study efficacy outcomes

The efficacy outcome measure will be assessed by both clinician-rated HAMD-24, Clinical Global Impression (CGI) Scale and self-rated PHQ-9. HAMD-24, a well-established representative scale for patient selection and follow-up assessments, has been selected to indicate the alteration in patients’ clinical symptoms both overall and in each subset item after treatment. When using this 24-item scale, a patient with the total score over 20 or higher will be considered to be suffering from moderate, severe or very severe depression [36–38]. This scale comprises 24 items indicating seven subsets includes: (1) anxiety/somatic symptoms; (2) weight alteration; (3) cognitive disorder; (4) diurnal symptom; (5) retardation; (6) insomnia; (7) helplessness and worthlessness and hopelessness [39]. The CGI is a 7-point scale, with a score based on the physician’s medical knowledge and experience regarding the patient’s general psychiatric condition. It includes the evaluation of the illness severity from 0 (normal) to 7 (the most severely ill) prior to therapy and two more aspects which are the therapeutic response and change from 1 (very much improved) to 7 (very much worse) after receiving medical treatment [40]. The Patient Health Questionaire-9 items (PHQ-9) is a self-reported questionnaire range from 0 to 27, each of the nine questions can be scored from 0 (not at all) to 3 (almost every day) according to the presence and severity of depression over past 2 weeks. It categorizes depression severity according to scores of 0–4 (none to minimal), 5–9 (mild), 10–14 (moderate), 15–19 (moderately severe) and 20–27 (severe) [41]. The Side Effects Rating Scale (SERS) of Asberg assesses the unwanted effects accompanied by antipsychotic drugs weekly, questions around the severity of increased fatigability, headache, insomnia, dizziness, orthostatic syncope, palpitations, tremor, increased tendency of sweating, reduced salivation, constipation, urination disorder, sleepiness, sexual dysfunction, and others. Its scores ranges from 0 (absent) to 3 (among the most severe) [42].

### Primary efficacy outcome measures

The reduction rate of the HAMD 24 score both in overall and in each subset item is defined as (Scores of each evaluation time point − baseline scores)/Baseline scores.

### Secondary efficacy outcome measures

- ■ Changed quantitative value, obtained by subtracting the HAMD-24 score at each evaluation time point from the baseline score
- ■ The changes in scores from baseline of other efficacy scales, such as the PHQ-9 and the CGI Scale

### Safety outcome measures

**Fig. 2:**
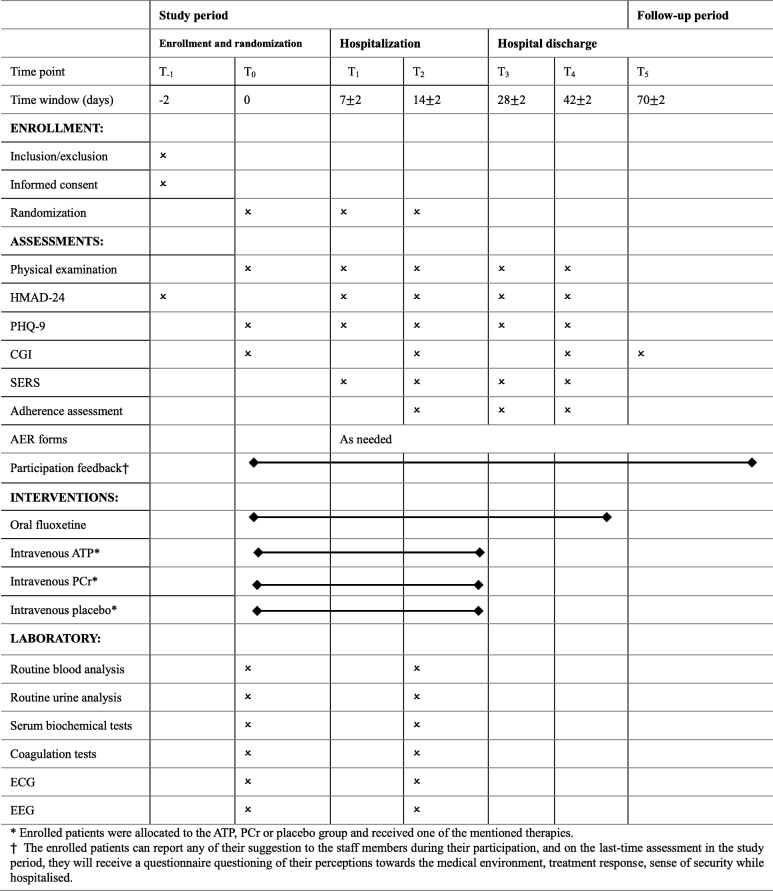
Participant timeline

- ■ The physical examinations (including vital signs such as blood pressure, temperature, pulse, respiratory rate, system review of heart, lungs, gastrointestinal, central nervous system and psychiatric status) and SERS will be conducted on each evaluation time point. (see Fig. 2)
- ■ Data from laboratory examinations, including routine blood and urine tests, serum biochemical tests (kidney function, liver function, proteins, iron status, and coagulation tests, as well as from instrumental examinations, such as electrocardiogram (ECG) and electroencephalogram (EEG), will be collected based on the schedule. (see Fig. 2)
- ■ If any adverse events occur, physicians will document them on the adverse events report (AER) forms and on the CRF. The side effects will be documented on the CRF as well

### Participant timeline

See Fig. 2.

### Sample size

Considering that there are no previous studies from which to perform sample size calculation, and assuming drop-out rate less than 15%, we anticipate enrolling 14 patients per arm for a final sample size of at least 12 per arm [43]. Our collected data will aid in the calculation of the appropriate sample size for further large-scale study.

### Recruitment

Patients with MDD will be recruited 1 year at Zhujiang Hospital, Southern Medical University, from May 2017 to April 2018. Posters for recruitment will be posted in the in-/out-patient buildings of the hospital and in the Mental Health Center of Southern Medical University. Network advertisements will be issued in public via social media circles. Generally, participants will contact us and set appointments prior to the screening. Afterward, two assigned physicians will evaluate whether participants are eligible for our study following the inclusion/exclusion criteria and will provide detailed information about our study if qualified; otherwise, relevant medical advice will be offered.

### Randomization and allocation concealment

Forty-two patients will be randomized at a ratio of 1:1:1. A list of random numbers, generated by Statistical Analysis Software (SAS), version 9.2, using the permuted block-randomization method, will be used to assign the participants to one of the three treatment groups. The randomization codes will be sealed in consecutively numbered opaque envelopes. An independent bio-statistician will conduct the above procedure, and the envelopes will be locked in a cabinet. The key will be held by a research nurse. After a patient is included in our study, each envelope will be opened in ascending order by the research nurse and assign their treatment accordingly.

### Blinding

Referring to the randomization code, the research nurse will be aware of the patients’ treatment assignments and will prepare the drugs before administration. The research nurse will refrain from discussing the treatment assignment with both researchers and patients as mentioned in the confidentiality agreement. In addition, the clinical researchers, bedside nurses who dispense drugs and participants will be blinded. To achieve blinding, study drugs or placebo will be labeled with 0.9% saline (110 ml) regardless of their actual assignment while intravenous infusion. Reversal of blinding will be permissible under the emergency condition of severe adverse incidents; otherwise, un-blinding will be performed after our final database is locked.

### Data management and monitoring

The clinical researchers will receive 2 weeks of trial-related training regarding the research content and scale instructions prior to the start of our study. All researchers have completed their courses in Good Clinical Practice. The rater for the HAMD-24 pair, CGI scale, and SERS are licensed and experienced physicians with psychiatric learning backgrounds. The majority of HAMD-24 scale assessment data will be collected by face-to-face joint interviews, with one researcher using the structured interview guide to initiate discussion with the patients, while the other researcher obtains the missing required information if necessary and scores it separately afterward; this procedure is intended to increase the data reliability. After grading, if any controversial items appear, a consensus conference will be held or the patients may even be reassessed on the same day to improve data accuracy. Interviews through video teleconference will be used in some exceptional situations. The CGI scale data will be collected according to the available information through the interview with patient, care-giver, or daily observation while hospitalized. The SERS will be scored based on the information provided by the patients and excludes the symptoms of somatic anxiety from MDD. The self-rated scale PHQ-9 will ensure the patients’ full comprehension of each question in advance. Laboratory tests and instrumental examinations will be conducted in the relevant hospital department. Collected patient data will be recorded on the CRF by assigned clinical researchers. An independent Data Monitoring Committee (DMC) will meet after a group of six participants has completed their final visits to review the ongoing recruitment and protocol compliance, to safeguard the interests of the trial participants, to assess the safety and efficacy of the interventions during the trial and the consistency between the original data and CRF, to ensure that every adverse event is recorded on AER forms and that they are securely stored and to monitor the overall conduction of the clinical trial. An independent clinical research associate will prepare a final report for the principle investigators (PIs), Dr. Lianxu Zhao and Prof. Tianming Gao after the visit.

### Statistical analyses

The statistical analyses mentioned below will be performed by a bio-statistician not affiliated with our study. Data from CRFs will be computed by two different researchers. Primary results will be based on the intention-to-treat principle. Data for feasibility and safety analysis will be presented as relative frequencies using descriptive statistics. The primary and secondary efficacy outcome measures will use analysis of covariance (ANCOVA) to compare the means between three groups at each time point after adjusting for the fixed factors (gender) and covariates (age, baseline scale score). Multiple comparisons between groups will be performed using the least significant difference (LSD) test. Repeated measures analysis of variance will be performed to determine whether the treatment effects changed over time. The missing values of the efficacy outcome measurements will be managed using the last-observation-carried-forward method. Additionally, sensitivity analysis will be further performed using original data without replacing values missing from the efficacy outcomes.

### Harms

After initiating treatment, the participants will be encouraged to report any discomfort, and the physicians will record it on CRF and determine whether the discomfort is related to the study drugs. If any adverse events occurred, they will be documented on AER forms and entered into CRF, including the nature of the events, onset dates, causality with the study drugs, final diagnoses, severity, any given corrective managements, and clinical outcomes. Furthermore, physicians will provide expert care until the patients recover, the relevant expenses will be covered and financial compensation will be provided to patients through study funding. Severe adverse events will be reported to the hospital Ethics Committee, PI and sponsor within 24 h, and they will decide whether the study procedures should continue or cease based on the patients’ best interests.

### Ethics and dissemination

#### Research Ethics approval

The trial protocol and informed consent form adhere to the principles of the Declaration of Helsinki [44], and template advertisements were reviewed and approved by the Medical Ethics Committee of Zhujiang Hospital of South Medical University prior to the start of our study on 28 April 2017 (Ethics Reference Number: 2017-SJNK-001).

#### Protocol amendments

Any modifications to the protocol referring to the conduct of the study or potential benefits to the patient or that could affect patient safety should be reapproved by the Medical Ethics Committee and sponsor prior to implementation.

#### Consent or assent

To those who meet the requirements, a consent form will be offered, covering the study name, registered information, inclusion/exclusion criteria, treatment plan and obligations, possible drug-related side effects, and expenses during participation, and a further period will be provided for the patients to consider whether they will participate. Any doubts can be discussed and allayed before participation, ensuring unambiguous comprehension of our study. Participants will retain a copy signed by both parties.

#### Confidentiality

Participants’ study-related information, including personal identification data, medical history, and collected assessment materials, will be stored in a locked file cabinet at the study site, to which only assigned study researchers can be permitted access for data collection or analysis. The privacy of the participants will be guaranteed by the PI. Documents submitted to DMC, Zhujiang Hospital, Southern Medical University, will be identified by the allocated ID only. Additionally, electronic data records for statistical purposes will be secured with a password-protected access system.

#### Access to data

The PIs will have full access to the complete trial data, all of which can be retrieved for later verification and validation. Moreover, no trial-related documents will be destroyed without prior joint permission from the PI and sponsor.

#### Ancillary and post-trial care

Following the study timeline, at the final visit during our study period, the physician will recommend a post-trial treatment plan based on the patient’s current condition and will prescribe drugs with the patient’s consent.

#### Dissemination policy

Our research has been registered at ClinicalTrials.gov with the assigned ID of NCT03138681. All publications and representations related to our study should be authorized by the PI in advance. Full access to the detailed protocol and database of our study information can be discussed with the PI. After study completion, final results are expected to be published and presented at related conferences.

## Discussion

Multiple studies have revealed altered energy metabolism in brain regions. With the utilization of in-vivo phosphorous magnetic resonance spectroscopy, nucleoside triphosphate (NTP), which mainly derives from ATP, has shown to be decreased in depressed subjects [45]. Meanwhile, in MDD patients treated with either SSRI sertraline, or creatine, or thyroid hormones, which have been shown to increase brain bio-energetic metabolism, NTP levels are significantly increased in treatment responders compared with non-responders [26, 46, 47]. Compared with conventional ADs, novel compounds with fast-acting AD effects will minimize the time lag to obtain treatment results, enhance patients’ adherence, reduce the suicide rate and aid psychosocial recovery.

Our preclinical studies showed that ATP, as well as PCr, displayed rapid AD effects in animal models of depression. To our knowledge, our study is the first clinical attempt to add ATP/PCr intravenously with the orally administered fluoxetine to patients with moderate to severe MDD.

PCr, after being diluted in 0.9% saline 100 ml, should be infuse intravenously within the next 40 min. As this is a double-blinded experiment, both ATP and placebo group should be administered in less than 40 min. However, if the infusion rate of ATP is too fast, some patients might experience palpitations. We assume that the nocebo effects might influence the efficacy results in ATP group.

After enrollment, patients will be hospitalized for the initial 14 days, enabling early detection, screening and treatment for adverse events when they occur, minimizing patient concern about the therapy, and enhancing drug adherence. Enrolled patients will be registered as a regular hospitalized patient, and be put in a regular two-/three-bed ward. The interaction influences among patients sharing the same ward will be evaluated through a questionnaire received in last visit of study period. However, compared with those suffering from physical diseases, most patients with mental illnesses including MDD are reluctant to be hospitalized, which adds to the recruitment difficulties. Negative family interactions, and social milieu could be unfavorable for recovery from depression [48]. Hospitalization removes patients from a real-world setting. Therefore, the isolation of passive psychosocial influences or, instead, safe surroundings and clinical care, might exert beneficial effects on MDD patients. However, those with welcoming, supportive supervised housing may return home after day treatment. The tailored hospitalization setting, whether they stay in the ward for the full day or only during the daytime, is performed according to the specific characteristics of the enrolled subjects’ both personality and environmental factors for the purpose of providing a patient-centered treatment setting [49]. We will examine the impact of this setting and discuss in the final report.

The small sample size of this pilot study does not allow us to observe significant statistical differences of efficacy outcomes among groups. However, existing trends of efficacy as well as the feasibility assessment will help determine whether to conduct a powered, full-scale trial and help appropriate sample size calculation.

### Trial status

Recruitment of participants is currently ongoing.

## Additional file

Additional file 1: Standard Protocol Items: Recommendations for Interventional Trials (SPIRIT) 2013 Checklist: recommended items to address in a clinical trial protocol and related documents*. (DOC 124 kb)

## Abbreviations

ADs: Antidepressants
AER: Adverse events report
ANCOVA: Analysis of covariance
ATP: Adenosine triphosphate
CGI: Clinical Global Impression
CK: Creatine kinase
CoQ10: Coenzyme Q10
CRF: Case Report Form
DMC: Data Monitoring Committee
ECG: Electrocardiogram
EEG: Electroencephalogram
HAMD: Hamilton Depression Rating Scale
IV: Intravenous
LSD: Least significant difference
MDD: Major depressive disorder
NMDA: *N*-methyl-D-aspartate
NTP: Nucleoside triphosphate
OXPHOS: Oxidative phosphorylation
PCr: Phosphocreatine
PFC: Prefrontal cortex
PHQ-9: Patient Health Questionaire-9 items
PIs: Principle investigators
SAS: Statistical Analysis Software
SERS: Side Effects Rating Scale
SFDA: State Food and Drug Administration
SSRIs: Selective serotonin reuptake inhibitors
